# Diabetes risk status and physical activity in pregnancy: U.S. BRFSS 2011, 2013, 2015, 2017

**DOI:** 10.1186/s12884-020-03434-5

**Published:** 2020-11-30

**Authors:** Bethany G. Rand, Tammie M. Johnson, Samantha F. Ehrlich, Laurie Wideman, James M. Pivarnik, Michael R. Richardson, Michelle L. Stone, James R. Churilla

**Affiliations:** 1 University of North Florida, Jacksonville, FL USA; 2 Florida A&M University, Tallahassee, FL USA; 3 University of Tennessee, Knoxville, TN USA; 4 University of North Carolina at Greensboro, Greensboro, NC USA; 5 Michigan State University, East Lansing, MI USA

**Keywords:** Pregnancy, diabetes, gestational diabetes, hyperglycemia, physical activity, muscle strengthening

## Abstract

**Background:**

Pregnant women without complications are advised to engage in physical activity (PA) to mitigate adverse outcomes. Differences may exist among pregnant women of diverging diabetes status in meeting national PA recommendations. We sought to examine differences in aerobic activity (AA) and muscle strengthening activity (MSA) by diabetes risk status (DRS) among pregnant women in the United States.

**Methods:**

The sample (*n* = 9,597) included pregnant women, age 18–44 years, who participated in the 2011, 2013, 2015, and 2017 Behavioral Risk Factor Surveillance System. Levels of DRS include: no diabetes (ND), high risk for diabetes (HRD) due to self-reported gestational diabetes or pre-diabetes, and overt diabetes due to self-reported, clinically diagnosed diabetes (DM). Odds ratios (ORs) and 95% confidence intervals (CI) for meeting PA recommendations were obtained. Covariates included age, race, education, household child count, alcohol consumption, and smoking status.

**Results:**

Findings revealed that on average, DM had 46.5 fewer minutes of weekly AA compared to ND. Furthermore, a significantly lower OR (0.39; CI 0.19–0.82) for meeting both recommendations was observed in DM as compared to ND after adjustment.

**Conclusions:**

We observed that pregnant women with overt diabetes had a lower odds of engaging in PA, while those at high risk were similar in their PA engagement to ND. Future studies aimed at assessing determinants of PA behavior may help guide efforts to promote exercise in pregnant women with diabetes.

## Background

Hyperglycemia generally refers to the presence of higher than normal glucose levels in the blood [1]. In pregnancy, hyperglycemia may be due to chronic conditions such as type 2 diabetes mellitus (T2DM) or prediabetes (PD), or gestational diabetes mellitus (GDM). These three manifestations of hyperglycemia differ in their diagnostic criteria and severity. The United States (U.S.) prevalence of T2DM and PD in women aged 20 years and over has increased by at least two percentage points from 1999 to 2012, climbing to 13.8% and 35.9%, respectively [2]. The estimated prevalence of GDM in the U.S., based on data from the 2007–2014 National Health and Nutrition Examination Survey (NHANES) is 7.6% [3].

A T2DM diagnosis can be confirmed by: fasting plasma glucose (PG) ≥ 7 mmol/L, a two-hour (2-H) PG ≥ 11.1 mmol/L after a 75 g glucose load during oral glucose tolerance test (OGTT), or a glycohemoglobin (A1C) ≥ 6.5% [4]. Accurate diagnosis requires at least two separate positive readings for the same test. In addition, one instance of classic symptoms of hyperglycemic crisis with a random PG ≥ 11.1 mmol/L may confirm diagnosis. Type 2 diabetes diagnosis heightens the risk for blindness, kidney failure, lower limb amputations, cardiovascular events, and complications in pregnancy [5]. Type 2 diabetes has also been shown to augment risk for cardiovascular diseases (CVD) [6].

Prediabetes diagnosis is like that of T2DM, modified with lower cut points: fasting PG 5.6–6.9 mmol/L, 2-H OGTT 7.8–11.0 mmol/L after a 75 g glucose load, and A1C 5.7–6.4% [1]. Not unlike T2DM, PD carries a risk of damage to the eyes, kidneys, blood vessels, and heart [7]. Furthermore, 5–10% of patients with PD progress to T2DM annually [8]. In order to prevent disease progression, first line treatment includes: weight loss of 5–10% of body weight and 30 minutes a day of moderate intensity physical activity (PA).

Gestational diabetes initiates in pregnancy and resolves after delivery [2]. Diagnosis is often based on a 3-H 100 g OGTT. Diagnosis is confirmed by two or more of: a fasting PG 5.3–6.9 mmol/L, 1-H PG ≥ 10.0 mmol/L, a 2-H PG 8.6–11.0 mmol/L, and a 3-H PG 7.8–11.0 mmol/L [9]. However, screening methods and diagnostic criteria have varied across years and governing bodies [10]. This has led to varying prevalence estimates and uncertainty for patients who may not have received GDM diagnosis in previous years[11]. Although GDM is not a lifelong disease, it is associated with over a seven-fold risk for T2DM [12] and a 50% increased risk for CVD [13]. Maternal and fetal sequelae of GDM include increased perinatal mortality, fetal macrosomia, neonatal hypoglycemia, cesarean section, and postpartum depression [14]. Furthermore, glucose intolerance, T2DM, and obesity risk are heightened in GDM offspring[15].

Physical activity has been shown to restore insulin sensitivity and minimize impaired glucose tolerance in pregnancy [16]. A meta-analysis of 40 observational studies reported a 30% reduction in GDM risk for any general amount of PA[17]. Further evidence of the benefits of PA comes from a 2020 meta-analysis by Doi et al., which reported a 30% GDM risk reduction as the overall effect of 11 PA interventions in pregnancy [18]. Exercise can positively impact fetal body composition with an overall increase in fetal weight and decrease in percent of fetal mass. This is due to improved maternal glucose control, improved maternal autonomic control, improved placental oxidative stress, and placental efficiency [19].

In 2008, the Department of Health and Human Services (DHHS) provided PA recommendations for the health and well-being of American citizens, pregnant women included, with new, revised recommendations released in 2018 [20]. The new 2018 PA Guidelines for adults are comparable to the previous guidelines modified to allow AA bout duration minimums of 2 minutes [21]. Although national guidelines have been established, clinical advice on PA may differ across the U.S., depending on the personal views, time constraints, training, and confidence in dissemination of PA recommendations [22, 23].

Due to their unique medical considerations, pregnant women have separate recommendations for PA. Current recommendations made by the American College of Obstetricians and Gynecologists (ACOG) in 2020 state that exercise and/or PA is beneficial for most pregnant women but modifications in exercises may be necessary to account for physiological and anatomical changes [24]. Pregnant women should be thoroughly evaluated by an obstetrician-gynecologist before PA recommendations are made to ensure the patient does not have medical contraindications. Women with uncomplicated pregnancies should be encouraged to engage in aerobic and muscle strengthening activities (MSA) before, during, and after pregnancy. Furthermore, activity restriction should not be routinely prescribed as a treatment to reduce preterm birth. Indeed, sedentary behavior has shown to be associated with increased risk of GDM despite high PA levels, and particularly in women with excessive gestational weight gain [25].

The 2008 and more recent 2018 U.S. DHHS guidelines on PA in pregnancy recommend at least 150 minutes of moderate-intensity AA per week, avoiding supine position and high fall risk sports such as horseback riding [20, 21]. Similarly, the 2019 Canadian guidelines recommend 150 minutes of moderate-intensity aerobic activity (AA) per week, a minimum of three days per week [26]. Reinforcement of the importance of these recommendations comes from a recent umbrella review which found strong evidence that moderate-intensity PA reduced the risk of GDM, symptoms of postpartum depression, and excessive gestational weight gain [27].

In addition, Canadian guidelines encourage incorporation of a variety of aerobic and resistance exercise in addition to yoga, stretching, and pelvic floor muscle training. Limited evidence exists on ideal dose of MSA for pregnant women [28]. However, as previously mentioned, resistance exercise is encouraged in both Canadian and ACOG guidelines [24, 26].

Despite the overwhelming evidence of benefits [29], less than 15% of women achieve the minimum recommendation of 150 minutes of moderate intensity PA per week during pregnancy [30]. About one third of pregnant women do not engage in any PA [31]. Understanding the various characteristics and behaviors which may contribute to PA engagement or lack thereof is necessary to inform effective interventions. Such factors include proxies for social determinants of health such as race/ethnicity and education level, characteristics such as age and number of children [32–35], and behaviors such as smoking and pre-pregnancy PA levels [34, 35].

Though we know that PA recommendations in pregnancy are infrequently met, sparse information exists on how self-reported GDM and PD histories compare with self-reported diabetes and euglycemia in meeting AA recommendations and two days of MSA per week. This study will examine the differences in PA engagement for parous women with varied diabetes risk status (DRS). Therefore, the study aims to answer three questions: (1) Is there an association between DRS and meeting the 2008 DHHS PA recommendation in pregnancy? (2) Is there an association between DRS and engaging in at least two days of MSA per week in pregnancy? (3) Are there other major characteristics that are associated with meeting the AA recommendations and two days of MSA in pregnancy?

## Methods

### Sample Population

The data come from the 2011, 2013, 2015, and 2017 Behavioral Risk Factor Surveillance System (BRFSS), a population-based survey administered through random-digit-dialed landline and cellular telephones. The BRFSS obtains information on participant demographics, health behaviors, and health related issues. Data are collected on the noninstitutionalized U.S. civilian population in all 50 states, the District of Columbia, and three U.S. territories. Sections were stratified according to state regions and within each stratum are randomized cluster units (households). The raking method for sample weighting was used to ensure appropriate representation of demographic variables. This method allows inclusion of education level, marital status, renter/owner status, gender, age, and race/ethnicity, and landline or cell phone in the final weights. Each dimension is adjusted separately as an iterative process, which can continue up to 75 times or until data converges to Census estimates. Participants are pregnant women between the ages of 18 and 44 who completed all relevant sections of the BRFSS. Women who reported a diabetes diagnosis at age 5 or younger were excluded, as they were likely to have type 1 diabetes. After excluding incomplete responses and probable type 1 diabetes (*n* = 1,482), there was a total of 9,597 participants.

### Independent Variable

To obtain the independent variable, DRS, participants were asked if they had ever been told by a doctor that they had diabetes and whether it was only when they were pregnant. Women reporting “yes” to this question were given diabetes status. Those who reported diabetes only in pregnancy or prediabetes were classified as GDM and PDM, respectively, and considered at a high risk for T2DM. Those who reported having no diabetes were considered to have non-diabetes status. Therefore, three DRS groups were established: high risk for diabetes (HRD; n = 457), no diabetes (ND; n = 9036), and diabetes (DM; n = 104).

### Dependent Variables

The dependent variables in this study were engaging in AA, MSA, both, and neither recommendations based on the 2008 DHHS guidelines. To obtain the AA variable, participants were asked about the type, frequency, and duration of weekly PA performed in the past month. Depending on the intensity and total minutes of AA, participants either met or did not meet the AA guidelines. Additionally, minutes of AA were examined as a continuous variable. The frequency of MSA was obtained by participants being asked the question: “During the past month, how many times per week or per month did you do physical activities or exercises to strengthen your muscles?” Depending on the frequency of MSA (less than two times per week or at least two times per week), participants either met or did not meet the MSA guidelines.

### Characteristics

Estimates of association between DRS and PA were adjusted for age, race, level of education completed, number of children in the household, alcohol consumption, and smoking status. Table 1 breaks down the categorization of each variable. These adjustment variables were selected a priori, based on previous literature. A sensitivity analysis that included additional adjustment for calendar year yielded identical results (data not shown).

**Table 1:** Characteristics of Pregnant Women by Diabetes Risk Status:BRFSS 2011, 2013, 2015, 2017

	Total	ND	HRD	DM	χ^2^ Test
	N (Weighted%)				P
**Total**	*N* = 9597	9036 (94.3)	457 (4.8)	104 (0.9)	
**Age**					< 0.0001
18–24	2113 (28.8)	2033 (29.7)	53 (11.6)	27 (31.7)	
25–29	2713 (27.2)	2592 (27.3)	96 (25.5)	25 (23.1)	
30–34	2873 (27.9)	2688 (27.5)	162 (37.0)	23 (22.5)	
35–39	1485 (12.4)	1351 (12.0)	115 (19.8)	19 (14.1)	
40–44	413 (3.7)	372 (3.5)	31 (6.1)	10 (8.6)	
**Race/Ethnicity**					0.1571
White	6203 (51.7)	5882 (52.0)	269 (46.1)	52 (54.8)	
African American	825 (12.9)	779 (13.1)	34 (8.2)	12 (10.8)	
Native American/Alaskan	242 (1.3)	224 (1.3)	14 (0.9)	4 (0.6)	
Asian	342 (5.9)	317 (5.7)	21 (8.8)	4 (8.6)	
Native Hawaiian/ Pacific Islander	88 (0.3)	78 (0.2)	7 (0.8)	3 (0.5)	
Hispanic	1584 (26.0)	1463 (25.6)	96 (33.0)	25 (21.2)	
Other	313 (1.9)	293 (1.9)	16 (2.1)	4 (3.4)	
**Education Level**					0.1948
Did not complete HS	777 (16.2)	710 (15.8)	56 (22.9)	11 (15.4)	
Completed HS	2176 (24.2)	2030 (24.1)	113 (26.1)	33 (27.9)	
Some college/technical school	2553(28.9)	2403 (29.2)	122 (24.8)	28 (22.7)	
Graduated college/technical school	4091 (30.7)	3893 (30.9)	166 (26.1)	32 (34.0)	
**Number of Children in Household**					0.0024
None	2981 (33.1)	2862 (33.8)	87 (19.8)	32 (38.5)	
1–3 children	5979 (60.2)	5594 (59.7)	324 (72.0)	61 (51.4)	
4 or more	637 (6.6)	580 (6.5)	46 (8.2)	11 (10.1)	
**Alcohol Consumption (Based on the past 30 days)**					< 0.0001
None	8606 (88.8)	8091 (88.6)	429 (93.5)	86 (78.4)	
Moderate	883 (9.2)	848 (10.0)	23 (5.2)	12 (14.8)	
Heavy	108 (1.1)	97 (1.4)	5 (1.3)	6 (6.8)	
**Smoking Status**					0.1091
Never smoker	6758 (70.3)	6398 (71.7)	292 (64.9)	68 (68.0)	
Former smoker	2012 (21.0)	1864 (19.6)	126 (27.7)	22 (20.1)	
Current smoker	827 (8.7)	774 (7.3)	39 (7.4)	14 (11.9)	

*BRFSS* Behavioral Risk Factor Surveillance System; *ND* no diabetes; *HRD* high-risk for diabetes due to self-reported gestational diabetes or prediabetes; *DM* overt diabetes; *HS* high school; level of significance set to *P* ≤ 0.05

### Statistical Analysis

Data was analyzed with SAS version 9.4. Variables of interest were re-coded, and prevalence estimates were stratified by DRS using PROC SURVEYFREQ. All procedures included the sample weight, strata, and cluster variables to account for the complex stratified sampling design of BRFSS. PROC SURVEYMEANS was used to determine mean frequencies for continuous variables. Chi-square (*X*^2^) tests for equal proportions were used to check for statistical significance (P ≤ 0.05). Normality was checked and medians obtained with PROC UNIVARIATE.

Beta estimates (B) for the continuous AA variable were obtained using the SURVEYREG procedure. There was a non-normal distribution for the continuous AA variable, but the sample size was large enough to allow for linear regression without violations. All variables were then converted to categorical or dichotomous, with aerobic PA, MSA, both, and neither dichotomized into “meets recommendations” or “does not meet recommendations”. The SURVEYLOGISTIC procedure allowed for attainment of odds ratios (ORs) and 95% confidence intervals (CI) related to the proposed research questions. Furthermore, standardized beta coefficients (STB) were produced. By examining the absolute value of the STB and the P-value for statistical significance, characteristics controlled for in the final model were ranked for their contribution to each dependent variable.

## Results

Table 1 illustrates proportions for sample population characteristics in the total sample and stratified by self-reported DRS (i.e. ND: reporting never been told they have diabetes, DM: have been told they have diabetes, and HRD: have only been told they have diabetes in pregnancy or pre-diabetes). Statistically significant variance in distributions between DRS categories are observed for age, number of children in the household, and alcohol consumption. Apart from meeting the MSA recommendation, Table 2 illustrates the general pattern of decreasing prevalence of PA from ND to HRD to DM.

**Table 2:** Prevalence Estimates for Physical Activity According to Diabetes Risk Status: BRFSS 2011, 2013, 2015, 2017

Diabetes Status	No AA	No MSA	Meets AA Rec^a^	Meets MSA Rec^b^	Meets Both Rec^c^	Meets Neither^d^
	**n (weighted %)**					
**ND** ***N* = 9036**	2525 (27.9%)	6265 (69.3%)	3709 (39.2%)	1681 (16.9%)	1087 (10.9%)	4733 (54.8%)
**HRD** ***N* = 457**	150 (32.8%)	358 (78.3%)	182 (39.2%)	60 (15.7%)	42 (10.5%)	257 (55.6%)
**DM** ***N* = 104**	65 (62.5%)	80 (76.9%)	36 (30.1%)	13 (17.9%)	7 (5.0%)	62 (57.0%)
**Total*N* = 9597**	2720 (28.3%)	6674 (69.5%)	3927 (39.2%)	1754 (16.8%)	1135 (10.8%)	5052 (54.8%)

*BRFSS* Behavioral Risk Factor Surveillance System; *ND* no diabetes; *HRD* high-risk for diabetes due to self-reported gestational diabetes or prediabetes; *DM* overt diabetes; *AA* aerobic activity; *MSA* muscle strengthening activity; ^a^2008 Department of Health and Human Services (DHHS) recommendation of 150 minutes of moderate intensity AA/wk.^b^2008 DHHS recommendation of 2 days/wk of MSA. ^c^both “a” and “b”; ^d^neither “a” nor “b”; level of significance for *X*^2^ test set to *P* ≤ 0.05; Chi-square tests were statistically significant (*P* < 0.05) for all measures.

### Differences in Aerobic Activity by Diabetes Risk Status

Table 3 provides B values for minutes of AA per week in the HRD and DM groups (ND referent) for crude, age adjusted, and fully adjusted models. In the adjusted model, those with diabetes had 46.5 fewer minutes in AA compared to those with no diabetes. ​Having HRD contributes modestly to the likelihood of engaging in AA when compared to having no diabetes.

**Table 3:** Linear Regression for Aerobic Activity in per Week in Pregnancy by Diabetes Risk Status

	HRD	DM
***Model***	**B (SE)**	**B (SE)**
**Crude**	-23.6 (0.041)	-45.9 (0.041)
**Age Adjusted**	-22.2 (0.036)	-43.4 (0.320)
**Fully Adjusted**^**a**^	-2.51 (0.078)	-46.5 (0.078)
^a^Accounts for age, race/ethnicity, education level, number of children in the household, alcohol consumption, and smoking; *B* beta regression estimate; *HRD* high risk for diabetes due to self-reported gestational diabetes or prediabetes; *DM* overt diabetes; *SE* standard error *P* < 0.0001 level of significance for all values listed		

### Odds Ratios for Meeting PA Guidelines in Pregnancy by DRS

Table 4 represents odds of meeting AA, MSA, both, and neither 2008 DHHS recommendations. After adjustments, the odds of meeting both AA and MSA recommendations were approximately 60% lower in the DM group (ND referent; OR 0.39; CI 0.19–0.82). No other statistically significant relationship between DRS and PA recommendations was observed.

### Differences in Muscle Strengthening Activity

Interestingly, although the odds of meeting both recommendations were significantly lower in group DM compared to group ND (Table 4), the prevalence of MSA was slightly higher (Table 2). Not illustrated are the median number of days of MSA per week in women reporting at least one day of MSA in the past 30 days: 2.00, 2.00, and 1.00 in groups ND, HRD, and DM, respectively. Table 5 exhibits results from a subgroup analysis limited to only women who met the AA recommendations to determine whether the DM subgroup differ in meeting the MSA recommendations when compared to the ND and HRD subgroups. Although not statistically significant (*P* = 0.3382), the percentage of DM who met the MSA recommendations (16.5% SE 6.0%) was lower than ND and HRD percentages (27.8% and 26.9%, respectively), potentially clinically significant.

**Table 4:** Odds Ratios for Meeting Physical Activity Recommendations by Diabetes Risk Status

		Meets AA	Meets MSA	Meets Both	Meets Neither
**Crude**	HRD	OR 0.96 CI 0.66–1.40	OR 1.00 CI -0.58-1.72	OR 0.96 CI 0.44–2.08	OR 1.03 CI 0.72–1.49
	DM	OR 1.02 CI 0.53–1.95	OR 0.36 CI 0.11–1.12	OR 0.43* CI 0.2–0.91	OR 1.09 CI 0.64–1.86
**Age-adjusted**	HRD	OR 1.01 CI 0.70–1.48	OR 1.09 CI 0.60–1.98	OR 0.96 CI 0.44–2.10	OR 1.02 CI 0.71–1.47
	DM	OR 0.67 CI 0.39–1.17	OR 0.93 CI 0.44–1.99	OR 0.43* CI 0.20–0.92	OR 1.08 CI 0.64–1.8
**Fully adjusted**^**a**^	HRD	OR 1.07 CI 0.72–1.59	OR 1.15 CI 0.66-2.00	OR 1.23 CI 0.58–2.60	OR 0.93 CI 0.64–1.36
	DM	OR 0.64 CI 0.37–1.11	OR 1.00 CI 0.45–2.23	OR 0.39* CI 0.19–0.82	OR 1.15 CI 0.68–1.95

*P < 0.05 level of significance; ^a^Adjusted for age, race/ethnicity, education level, number of children in household, alcohol consumption, and smoking status; *AA* aerobic activity; *MSA* muscle strengthening activity; ^a^2008 Department of Health and Human Services (DHHS) recommendation of 150 minutes of moderate intensity AA/wk.^b^2008 DHHS recommendation of 2 days/wk of MSA. ^c^both “a” and “b”; ^d^neither “a” nor “b”; HRD: high risk for diabetes due to self-reported gestational diabetes or prediabetes; DM: overt diabetes Referent group: no diabetes

**Table 5:** Proportions of Pregnant Women Meeting the MSA^a^ Recommendations Among those who Meet the AA^b^Recommendation​

	ND​	HRD​	DM​
***n​***	1087​	42​	7​
***Percent​***	27.8​	26.9​	16.5​
***Standard Error​***	1.2​	7.9​	6.0​

*P* Value* ​= 0.3382​

*P value derived from Wald Chi-Square Test​; ^a^2008 Department of Health and Human Services (DHHS) recommendation of 2 days/wk of muscle strengthening activity; ^b^2008 DHHS recommendation of 150 minutes of moderate intensity aerobic activity/wk; *ND* no diabetes; *HRD* high risk for diabetes due to self-reported gestational diabetes or prediabetes; *DM* overt diabetes

### Characteristics of Physical Activity

Table 6 describes the top three characteristics associated with the odds of meeting the 2008 DHHS recommendations. These characteristics were ranked by the absolute value of their STB to show, in order, their contribution to meeting the AA, MSA, both, and neither recommendations. The odds of meeting the AA recommendation were predominantly negatively associated with self-reported African American, Hispanic, or Asian race. The odds of meeting the MSA recommendation were positively associated with consuming alcohol in the past 30 days and completing more than high school and negatively impacted by having 1–3 children at home. The odds of meeting both and neither recommendations were highly associated with a combination of the top three AA and MSA determinants.

**Table 6:** Top Characteristics Contributing to Meeting Aerobic Activity and Muscle Strengthening Activity Recommendations in Pregnancy ​

PA Recommendation​	Characteristic ​	STB ​	*P* value ​
***AA***^***a***^			
** 1 ​**	African American ​	-1.28 ​	< 0.0001 ​
** 2 ​**	Hispanic ​	-1.16 ​	0.0015 ​
** 3 ​**	Asian ​	-1.08 ​	0.0043 ​
***MSA​***^**b​**^			
.**1 ​**	Consumed Alcohol in Past 30 Days ​	2.00 ​	< 0.0001 ​
** 2 ​**	Completed > HS ​	1.90 ​	0.0002 ​
** 3 ​**	1–3 Children at Home ​	-1.62 ​	< 0.0001 ​
***Both ​***^***c***^			
** 1 ​**	1–3 Children at Home ​	-1.97 ​	< 0.0001 ​
** 2 ​**	Consumed Alcohol in Past 30 Days ​	1.75 ​	< 0.0001 ​
** 3 ​**	African American ​	-1.58 ​	0.016 ​
***Neither***^***d***^			
** 1 ​**	Consumed Alcohol in Past 30 Days ​	-1.40 ​	< 0.0001 ​
** 2 ​**	Hispanic ​	1.37 ​	0.0001 ​
** 3 ​**	African American ​	1.26 ​	< 0.0001 ​

*HS* High School​; *PA* physical activity; *AA* aerobic activity; *MSA* muscle strengthening activity; ^a^2008 Department of Health and Human Services (DHHS) recommendation of 150 minutes of moderate intensity AA/wk.^b^2008 DHHS recommendation of 2 days/wk of MSA. ^c^ both “a” and “b”; ^d^ neither “a” nor “b”; STB: Standardized beta coefficient; All variables included in the model were diabetes risk status, age, race, education level, number of children in household, alcohol consumption, and smoking status

### Trends in Physical Activity and Diabetes Risk Status: 2011–2017

**Fig. 1:**
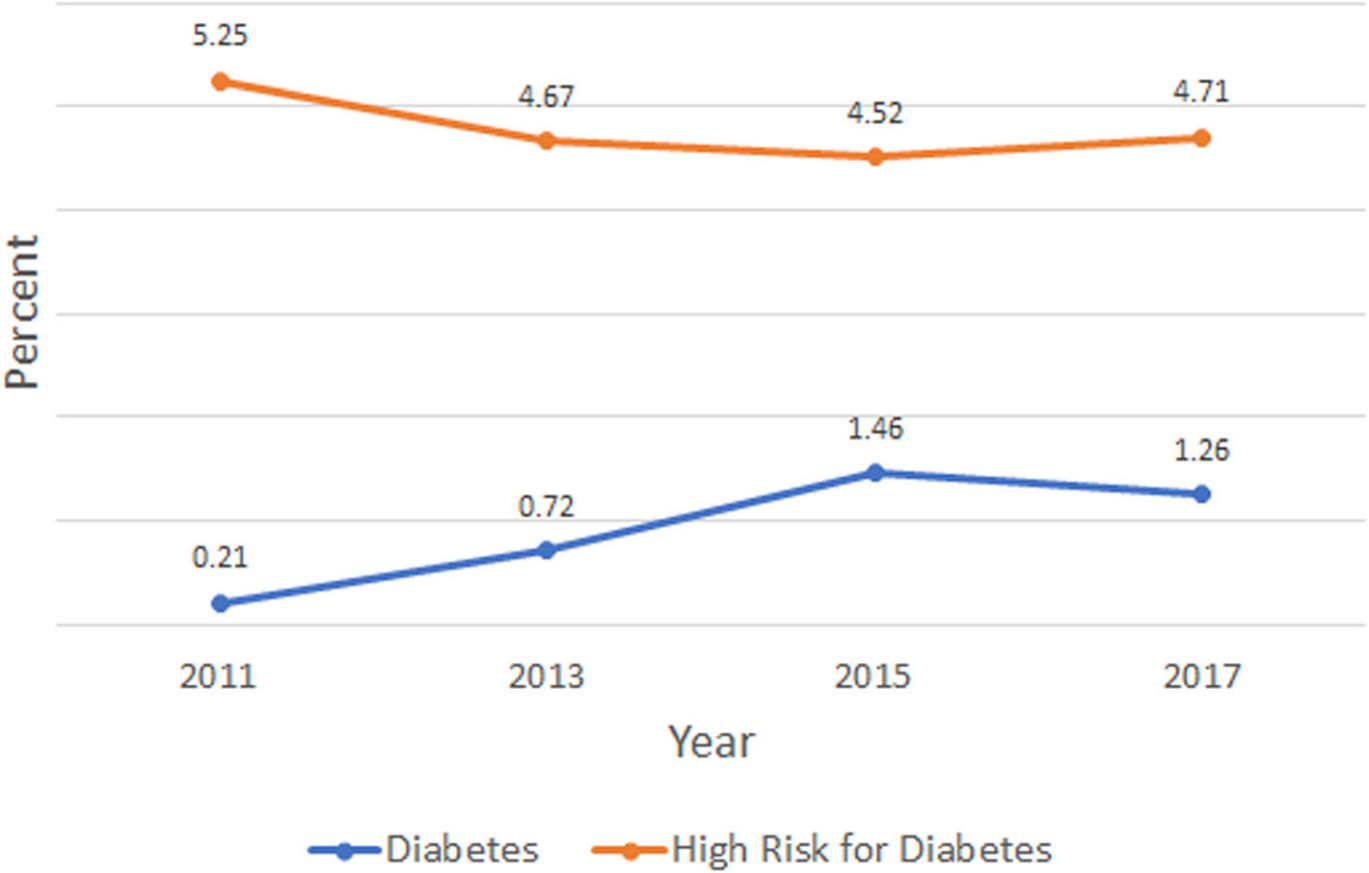
Trends in Prevalence of Overt Diabetes and High-Risk for Overt Diabetes in Pregnancy: BRFSS 2011-2017

From Table 7, we can see no statistically significant change in meeting 2008 DHHS PA recommendations (P > 0.05) across BRFSS interview years. However, although not statistically significant, the slight and consistent uptrend in MSA, from 15% in 2011 to 19% in 2017, should be noted. Furthermore, no significance was seen in distribution of DRS in pregnancy by interview year (Fig. 1), although a five-fold increase in overt diabetes prevalence from 2011 to 2017 may be observed.

**Table 7:** Prevalence of Meeting PA Recommendations in Pregnancy by Year ​

*Year* *n*	2011 (*n* = 2773)	2013 (*n* = 2638)	2015 (*n* = 2031)	2017 (*n* = 2155)	χ^2^ Test
***Recommendation​***	n (%) ​	n (%) ​	n (%) ​	n (%) ​	P-Value ​
**AA​**^**a**^	1122 (39.9) ​	1056 (38.1) ​	855 (37.8) ​	894 (40.7) ​	0.5954 ​
**MSA**^**b**^	438 (15.0) ​	469 (15.5) ​	403 (17.8) ​	444 (19.0) ​	0.1061 ​
**Both ​**^**c**^	284 (10.1) ​	295(9.8) ​	267 (11.6) ​	290 (11.7) ​	0.5129 ​

Percentages are weighted; ^a^2008 Department of Health and Human Services (DHHS) recommendation of 150 minutes of moderate intensity AA/wk. ^b^2008 DHHS recommendation of 2 days/wk of MSA. ^c^both “a” and “b”

## Discussion

This study aimed to examine the association between DRS and meeting the 2018 DHHS guidelines in pregnancy. Markedly observed in this study is the inverse association of overt diabetes and meeting both AA and MSA recommendations. This finding is reflective of a BRFSS study on nonpregnant women, reporting that non-pregnant women of a childbearing age with current diabetes are 40% more likely to fail to meet LTPA recommendations compared with their non-diabetes counterparts (*P* < 0.05) [30].

This study combines GDM and PD together as one high-risk for diabetes group. Though secondary to diabetes, both GDM and PD carry gravid and post-gravid health threats [7, 12–15, 36]. As shown in Fig. 1, the prevalence of overt diabetes in pregnancy increased from 2011 to 2015. This rise in prevalence may reflect improvement in diabetes management which has made child birth a realistic goal for women with diabetes [37].

Furthermore, relatively small sample sizes for DM and HRD groups may have contributed to a loss of statistical power to accurately demonstrate some relationships. With regards to meeting the AA guidelines, our study found no significant differences in ORs after adjusting for covariates in the fourth model. This mirrors previous findings from a 2003 BRFSS study examining nonpregnant women ages 18–44 (*n* = 4718), with and without a history of GDM where there was no difference in meeting the AA guidelines between groups after adjusting for age, race, education level, current employment, marital status, presence of children in household, smoking status, self-rated health, and BMI [35].

Top characteristics for odds of meeting the U.S. DHHS PA guidelines for adults closely mirrored differences in sample population characteristics. African American, Hispanic, and Asian race/ethnicities were negatively associated with odds of meeting AA recommendations. Although there was no statistical significance in the distribution (*P* = 0.1571), there may be intra-variability in these race/ethnicity categories. Specifically, 33% of the HRD group was Hispanic compared to 26% of the ND group and 21% of the DM group. Published evidence has identified Hispanic minority as major demographic risk factor for GDM, a large portion of the HRD group [15]. Furthermore, being part an ethnic minority is associated with higher diabetes prevalence [38].

We found that completing more than high school were positively associated with the odds of meeting the MSA recommendations while lower education level has been identified as a predictor for T2DM [38]. Having 1–3 children significantly contributed to higher odds of MSA. Having four or more children was not a top characteristic, most likely due to insufficient cell size. Previous literature has shown that having GDM and at least one child living at home were associated with compromised healthy lifestyle behaviors [35].

Given the widespread discouragement of alcohol consumption in pregnancy and deleterious effects of alcohol on fetal development [39], the strong positive contribution of alcohol consumption on odds of meeting MSA and both recommendations in pregnancy seems peculiar. However, alcohol consumption has been observed to favorably improve the odds of meeting MSA guidelines in adults with dyslipidemia and augmented waist circumference [40]. In another study examining the relationship between alcohol consumption and metabolic syndrome in adults, moderate and above moderate alcohol consumption was positively associated with improved metabolic factors, including decreased PG levels [41]. More research is needed to understand this relationship outside of pregnancy. However, existing evidence on the harmful effects of alcohol exposure on the fetus still warrant caution during pregnancy [39].

This study was not without its limitations. The cross-sectional nature of BRFSS does not allow us to infer causality. According to a 2015 CDC report, 31.1% of all U.S. women have PDM but only 14.1% are aware of their disease state [42]. Since our study relied on self-report, we may have mistakenly classified a large percentage of high-risk women as normal, which may have buffered the true influence of diabetes status on PA participation. Variables that may provide additional information when accounting for risk that were not included in the survey include pre-pregnancy BMI, specific diabetes subtypes, pre-conception care, and contraindications to exercise. In particular, the lack of information on gestational age hindered us from identifying women that may be overweight or obese and whether they were far enough along to be eligible for GDM screening/diagnosis. Furthermore, the study sample size did not allow examination of determinants of PA by DRS, due to unstable cell sizes.

Although MSA recommendations are not specified in the 2018 DHHS guidelines for pregnant women, we opted to include the MSA guidelines of two days of MSA per week in the general adult population. Evidence has demonstrated the role of strength training in diabetes prevention. To substantiate this, a prospective cohort study of non-pregnant women from the Nurse’s Health Study found that resistance exercise and lower intensity MSA were both associated with a lower risk of T2DM in the pooled analysis [43]. Greater glycemic load increased with greater volume of MSA, suggesting improved insulin sensitivity with this mode of activity. Resistance training has also been shown to ameliorate feelings of fatigue associated with pregnancy [44, 45]. More evidence is needed to identify potential differences in engaging in two days of MSA per week in addition to meeting the AA guidelines. However, based on our subgroup analysis, women with diabetes could po tentially be at the greatest deficit in accomplishing this. Based on our analysis and previous findings, focused effort should be made by clinicians to encourage muscle strengthening as a supplement to AA in women with overt diabetes during pregnancy.

Preconception counseling, with PA included, is recommended by the ADA [46]. Clinical recommendations to promote exercise in pregnant women with PD, GDM, and T2DM have been established [24, 46]. However, many women with diabetes are not meeting with clinical providers to receive prenatal counseling [47]. Moreover, cognitive dissonance may exist regarding healthy lifestyle and other lifestyle factors.

Furthermore, women in general may not be receiving quality exercise counseling by their physicians on exercise in pregnancy [48, 49]. Although PA guidelines are readily available to the public, few Americans are aware of what they are [50]. Evidence suggests that women are more likely to engage in PA if advised by their physician and therefore, it is essential that providers do so. [51]. Increased education by healthcare providers may also ameliorate feelings of uncertainty among certain women. Feeling unsafe/unsure about moderate PA may be associated with non-White race/ethnicity, low education, low income, and not participating in moderate PA with no intention to start exercising [52].

## Conclusions

Pregnancy is an opportunity for clinicians to encourage healthy lifestyle patterns, including PA. This study illuminates’ disparities in PA participation during pregnancy by diabetes status. Future studies should examine PA prevalence using objective measures of PA participation, hyperglycemia, and clinical assessment of participants. Ultimately, increased efforts should be made for interventions targeted at improving health outcomes by breaching the gaps in regular AA and MSA participation during pregnancy for women with DM, and characteristics such as multiple children, lower education, and/or racial/ethnic minority backgrounds, improving health outcomes.

## Data Availability

Annual BRFSS data is publicly available from the CDC at https://www.cdc.gov/brfss/annual_data/annual_data.html.
